# Heterogeneity and subtypes of CD4^+^ regulatory T cells: implications for tumor therapy

**DOI:** 10.3389/fimmu.2023.1291796

**Published:** 2024-01-05

**Authors:** Hanqing Lin, Yuanteng Xu, Chang Lin

**Affiliations:** ^1^ Department of Otolaryngology, Fujian Institute of Otorhinolaryngology, The First Affiliated Hospital of Fujian Medical University, Fuzhou, China; ^2^ National Regional Medical Center, Fujian Medical University, Fuzhou, China

**Keywords:** functional subtypes, tumor microenvironment, clinical trials, regulatory T cells, forkhead box protein 3

## Abstract

In the conventional view, CD4^+^ regulatory T cell (T_reg_) represents a subset of lymphocytes that involve the perception and negative regulation of the immune response. CD4^+^T_reg_ plays an important role in the maintenance of immune homeostasis and immune tolerance. However, recent studies have revealed that CD4^+^T_reg_ do not suppress the immune response in some diseases, but promote inflammatory injury or inhibit tissue remodeling, suggesting the functional heterogeneity of CD4^+^T_reg_. Their involvement in tumor pathogenesis is more complex than previously understood. This article reviews the relevant research on the heterogeneity of CD4^+^T_reg_, subtype classification, and their relationship with tumor therapy.

## 1 Introduction

CD4^+^ regulatory T cell (T_reg_) was first identified by Sakaguchi et al., who discovered a population of CD4^+^CD25^+^T cells in the thymus of mice that could suppress the progression of autoimmune diseases (1). Traditionally, CD4^+^T_reg_ has been believed can suppress the activities of T cells, B cells, natural killer (NK) cells, and other immune cells. Functional impairment of CD4^+^T_reg_ is a significant contributing factor to the development of autoimmune diseases (2). Studies in the field of tumor microenvironment (TME) have shown that tumor cells exploit the immunosuppressive capacity of CD4^+^T_reg_ in TME to evade immune surveillance and promote tumor progression (3). Therefore, targeting CD4^+^T_reg_ in tumor therapy holds significant potential for development.

Given the crucial role of CD4^+^T_reg_ in tumor progression, it is essential to gain a deeper understanding of the distinct functional subtypes of CD4^+^T_reg_. Recent advances in functional studies and single-cell sequencing have provided novel insights into the functional heterogeneity of CD4^+^T_reg_ (4, 5) and identified new molecular targets for CD4^+^T_reg_ in TME (6). This review aims to summarize the relevant research, including the functional heterogeneity and subtyping of CD4^+^T_reg_, and provide an overview of clinical trials targeting CD4^+^T_reg_.

## 2 Classification of CD4^+^T_reg_

CD4^+^T_reg_ can be classified into two main categories based on their cellular origins: natural-occurring T_reg_ (nT_reg_) and peripheral T_reg_ (pT_reg_). nT_reg_ are generated in the thymus, the precursor cells of T_reg_ mature into CD4^+^CD25^+^Foxp3^+^T_reg_ following stimulation by IL-2/STAT5 and IL-4 signals (7). On the other hand, pT_reg_ are derived from nT_reg_ or initial CD4^+^T cells in the periphery under specific cytokine or molecular stimuli (8). Additionally, there is a subset of CD4^+^T_reg_ known as induced T_reg_ (iT_reg_), which can be generated *in vitro* experiments. These three types of cells constitute the classical classification of CD4^+^T_reg_. Subsequent research has further refined this classical classification based on Foxp3 expression. For example, iT_reg_ can be further divided into Foxp3+iT_reg_ and Foxp3-iT_reg_, with the latter including Type I Treg (Tr1) cells (9), T helper 3 (Th3) cells (10), and IL-35-induced CD4^+^T_reg_ (iTr35) (11). **Table 1** summarizes the common types of CD4^+^T_reg_ and their corresponding phenotypes.

**Table 1:** Characteristic markers of different CD4^+^Treg subtypes.

Subtype of T_reg_	Phenotype	Effector molecules	Transcription factors	Reference
nT_reg_	CD4^+^CD25^+^Foxp3^+^Helios^+^CTLA4^+^ Nrp1^+^	TGF-β, IL-10, CTLA4, IL-35, LAG3, LAP, etc.	Foxp3	(12, 13)
Foxp3^+^iT_reg_	CD4^+^CD25^+^Foxp3^+^Helios^+^CTLA4^+^ Nrp1^-^	Similar to nT_reg_	Foxp3	(13)
Tr1	CD4^+^Foxp3^-^CD49b^+^LAG3^+^ CD226^+^	TGF-β, IL-10, PD-1, CTLA4, etc.	c-Maf, AhR, IRF4	(9)
Th3	CD4^+^CD25^–^CD69^+^Foxp3^–^LAP^+^TGF-β+	TGF-β	Not specified	(14)
iTr35	CD4^+^Foxp3^−^Ebi3^+^p35^+^IL-10^−^TGF-β^−^	IL-35	Not specified	(11)
T_reg_ induced by B cells	CD4^+^CD25^+^Foxp3^-^LAG3^+^ICOS^+⑦^PD1^+^GITR^+⑧^CD134^+^	IL-10, PD-1, CTLA4, LAG3, etc.	Not specified	(15)

## 3 Heterogeneity and subtypes of CD4^+^ T_reg_

### 3.1 Subtypes of CD4^+^T_reg_

Previous studies on CD4^+^T_reg_ have primarily focused on CD4^+^CD25^+^Foxp3^+^T_reg_. However, emerging evidence suggests functional heterogeneity within this population, and relying solely on the CD25^+^Foxp3^+^phenotype may lead to contradictory conclusions in studies investigating the same disease. Therefore, the establishment of a reliable subtyping strategy is crucial. Currently, two mainstream classification systems are widely used: functional subtypes proposed by the Sakaguchi et al. (16) and the Th-like T_reg_ subtypes based on cytokine expression (17).

Sakaguchi et al. proposed a subdivision of CD4^+^T_reg_ based on CD45RA expression in 2009, further categorizing them into three functional subsets: subset I consists of CD45RA^+^Foxp3^lo^/CD25^lo^ resting T_reg_ (rT_reg_); subset II comprises CD45RA-Foxp3^hi^/CD25^hi^ effector T_reg_ (eT_reg_), also known as activated T_reg_ (aT_reg_); and subset III includes CD45RA^-^Foxp3^lo^/CD25^lo^ non-T_reg_ (16). Functionally, rT_reg_ possess a certain degree of immunosuppressive capacity and express markers of naive cells such as CCR7 and CD62L. They can differentiate into eT_reg_ upon antigen stimulation. Subset of eT_reg_ exhibit stronger proliferation and immunosuppressive abilities but are more prone to apoptosis. Non-T_reg_, which were previously considered as cells secreting pro-inflammatory cytokines such as IFN-γ and IL-17 without immunosuppressive capabilities, were found to exhibit significant functional heterogeneity by Cuadrado et al. using mass spectrometry. CD127^+^ non-T_reg_ and CD127^-^ non-T_reg_ displayed characteristics of conventional T cells and eT_reg_, respectively. Furthermore, the CD127^-^ subset can be further subdivided based on CD49d and CCR4 expression, with CD127^-^CCR4^+^CD49d^-^ cells showing high levels of IL-2 expression, while CD127^-^CCR4^-^CD49d^+^ cells exhibit elevated levels of IFN-γ and IL-17 (18).

In addition to the classification proposed by Sakaguchi et al., Halim et al. categorized CD4^+^T_reg_ into four distinct subtypes, termed Th-like T_reg_, based on their cytokine secretion profiles and transcription factor expression in 2017. These subtypes include Th1-like T_reg_ (CCR4^+^CCR6^-^CXCR3^+^, primarily secreting IFN-γ and TNF-α), Th2-like T_reg_ (CCR4^+^CCR6^-^CXCR3^-^, primarily secreting IL-2, IL-4, IL-5, and IL-13), Th17-like T_reg_ (CCR4^+^CCR6^+^CXCR3^-^, primarily secreting IL-17A/IL-17F), and Th1/17-like T_reg_ (CCR4^+^CCR6^+^CXCR3^+^, secreting both IFN-γ and IL-17A without significant statistical differences compared to other subsets) (17). Each Th-like T_reg_ subset exhibited transcription factor expression and cytokine secretion patterns similar to their corresponding Th cell counterparts. Furthermore, all Th-like T_reg_ subtypes demonstrated immunosuppressive capabilities. However, this classification did not investigate the stability of Foxp3 expression in different subtypes, leading to inconsistent conclusions in various studies utilizing this classification system.

Besides to the aforementioned common classifications, the research conducted by Bluestone et al. revealed a stronger correlation between CD127 and Foxp3 expression levels compared to CD25. Furthermore, the suppressive function of the CD4^+^CD127^lo/-^ subset was found to be superior to that of the CD4^+^CD25^+^ subset (19). Currently, the phenotype of CD4^+^CD25^+^CD127^lo/-^ is widely used for isolation of CD4^+^T_reg_.

### 3.2 Foxp3 stability and the function of CD4^+^T_reg_

Current evidence suggests that stable expression of Foxp3 is closely associated with the immunoregulatory function of CD4^+^T_reg_. Therefore, the main factors regulating Foxp3 stability may be crucial for the heterogeneity of CD4^+^T_reg_ function, such as local inflammatory cytokine stimulation or epigenetic regulation.

Under stimulation from the inflammatory microenvironment, the expression of Foxp3 in some cells may be affected, leading to the loss of negative immune regulatory function. Early *in vitro* studies have shown that CD4+CD25- T cells can transiently express CD25 and Foxp3 upon IL-2 or TGF-β stimulation, but these T cells do not possess immunosuppressive abilities (20). Recent research by Yi et al. has found that, under the stimulation of the inflammatory cytokine IL-6, TIGIT-positive CD4^+^T_reg_ can stably express Foxp3 and maintain immunosuppressive function, while TIGIT-negative unstable cells lose Foxp3 expression and transition into an effector T cell phenotype (21). These results suggest that CD4^+^T_reg_ with stable Foxp3 expression may possess immune regulatory capabilities, while cells with unstable Foxp3 expression may transition into other types of T cells under microenvironmental influences.

In recent years, the role of epigenetic regulation in Foxp3 expression has become a new research focus, with DNA methylation being the most common modification. The gene *Foxp3* consists of 11 coding exons, 3 non-coding exons, and 10 introns. Within this gene region, there are 3 conserved non-coding sequences (CNS), with CNS2 containing numerous cytidine-phosphate-guanine (CpG) sites. These sites are methylated in effector T cells but exhibit demethylation in CD4^+^Treg cells (22), thus being referred to as the T_reg_-specific demethylated region (TSDR). Sakaguchi et al. previously discovered that there are differences in the methylation levels of the TSDR at the Foxp3 locus. TSDR in rT_reg_ and eT_reg_ exhibited demethylation, while TSDR in non-T_reg_ had a higher degree of methylation, suggesting that Foxp3 expression is more stable in rT_reg_ and eT_reg_ (16). Additionally, a recent study found that the CD28-PKC-NF-κB signaling pathway inhibits demethylation in the CNS2 region of peripheral CD4^+^T_reg_, causing unstable Foxp3 expression in pT_reg_ cells and subsequently affecting their immunosuppressive function (23). Furthermore, Ohkura et al. discovered a close relationship between single nucleotide polymorphisms in the epigenetic regulatory region of CD4^+^T_reg_ and susceptibility to autoimmune diseases (24). Apart from CNS2, two other CNS regions have been shown to play important roles in stable Foxp3 expression and immune tolerance: CNS1, located in the promoter region of the Foxp3 gene, was found to be associated with pT_reg_ differentiation and maintenance of fetal immune tolerance (25); and CNS3 mediates the clonal expansion of the CD4^+^T_reg_ receptor repertoire, thereby controlling excessive immune responses of autoreactive T cells and maintaining immune homeostasis (26). Additionally, research by Kitagawa et al. found that the transcription factor Satb1 can bind to a CD4^+^T_reg_-specific super-enhancer and a newly discovered conserved non-coding region upstream of the Foxp3 promoter prior to CD4^+^T_reg_ activation, promoting the development of CD4^+^T_reg_. This newly discovered region has been named CNS0 (27).

In addition to methylation, acetylation and histone modifications have also been shown to be associated with the stable expression of Foxp3. During the acetylation process, histone acetyltransferases (HATs) and histone deacetylases (HDACs) influence Foxp3 acetylation levels from different directions. Two studies by Li and Tao demonstrated that HAT TIP60 and class II HDACs (HDAC7 and HDAC9) can form complexes with Foxp3, regulating the immunosuppressive capacity of CD4^+^T_reg_ (28, 29). Other studies further investigated the impact of acetylation on CD4^+^T_reg_ function in specific diseases. Su et al. found that rheumatoid arthritis patients have a TIP60 functional deficiency, leading to instability of Foxp3 expression and impaired suppressive function of CD4^+^T_reg_ (30). Jiang et al. discovered that the acetyltransferase p300/CBP-associated factor (PCAF) can acetylate *Foxp3* and enhance its transcriptional activity, thereby promoting the suppressive function of CD4^+^T_reg_ (31). These studies suggest that acetylation and deacetylation processes play a critical role in regulating the stability and function of Foxp3 in CD4^+^T_reg_. In the field of histone modifications, substantial progress has been made in understanding the role of Enhancer of zeste homolog 2 (EZH2). EZH2 is a catalytic enzyme of the polycomb repressive complex 2 (PRC2) responsible for the methylation of histone H3 lysine 27 (H3K27) at the *Foxp3* locus, leading to the formation of H3K27me3 histone modification. Studies by DuPage et al. have demonstrated that Ezh2 is involved in stabilizing the expression of Foxp3 in a mice model, selective deletion of *Ezh2* in CD4^+^T_reg_ resulted in the development of autoimmune diseases and accompanied by reduced stability of CD4^+^T_reg_ (32). This study suggests that Ezh2 may play a crucial role in maintaining T_reg_ cell function. Goswami et al. found that selective deletion of *Ezh2* in CD4^+^T_reg_ delays tumor progression in mice, while the use of Ezh2 inhibitors in wild-type mice enhances the function of cytotoxic T lymphocytes (CTLs) and promotes the efficacy of anti-CTLA-4 antibodies, thus inhibiting tumor progression (33). These findings indicate that the histone modifications mediated by EZH2 may represent another important mechanism regulating the function of CD4^+^T_reg_.

## 4 Heterogeneity of CD4^+^T_reg_ in the TME

Infiltration of CD4^+^Tregs in TME suppresses local anti-tumor immune responses and is associated with poor prognosis in various types of cancer. The heterogeneity of CD4^+^T_reg_ offer diverse mechanisms that contribute to the modulation of anti-tumor immune responses and facilitate immune evasion. Therefore, accurate characterization of different CD4^+^T_reg_ subsets in TME is crucial for gaining insights into the formation of the immunosuppressive TME as well as for improving patient prognosis.

The classification of Tregs subsets proposed by Sakaguchi et al. have been widely used to study the heterogeneity of CD4^+^T_reg_ in TME. Saito et al., based on the classification, divided colorectal cancer into two types: Type A, characterized by predominant infiltration of eT_reg_ and correlated with poor prognosis, and Type B, characterized by predominant infiltration of non-T_reg_ and correlated with better prognosis (34). Similarly, studies on different types of tumors have consistently shown that eT_reg_ are the major population in TME and correlated with poor prognosis (35, 36). Wang et al. found a high overlap between the phenotypes and TCR repertoire of tumor-infiltrating CD4^+^T_reg_ and peripheral eT_reg_ in patients with breast cancer. They proposed a cytokine signal index (CSI) based on the responsiveness of various peripheral CD4^+^T_reg_ subsets to four cytokines, the CSI of eT_reg_ could effectively predict patient prognosis and relapse (37).

Limited research focused on Th-like T_reg_ in TME. Mizukami et al. have shown that tumor-infiltrating CD4^+^T_reg_ express CCR4 in lymphoma and gastric cancer (38). Halim et al. demonstrated that CCR4^+^T_reg_ in the TME predominantly exhibit Th2-like characteristics, they also discovered a significant elevation of CCR8 expression on Th2-like T_reg_ (17), consistent with the single-cell sequencing results reported by De Simone (39). Van Damme et al. also confirmed that tumor-infiltrating CCR8^+^T_reg_ was highly activated and immunosuppressive in both human and mouse tumors. Selective depletion of this subset resulted in increased responsiveness to immunotherapy (40). Downs-Canner et al. discovered that Th17 cells can differentiate into Th17-like T_reg_ in ovarian cancer, thereby suppressing immune responses (41).

The emergence of single-cell RNA sequencing technology has advanced our understanding of the functional subgroups of CD4^+^T_reg_ in tissue microenvironments, particularly in tumor-infiltrating sites. Several studies analyzed the immune cell infiltration in head and neck squamous cell carcinoma (HNSCC), non-small cell lung carcinoma (NSCLC), and breast cancer, respectively. Cillo et al. categorized CD4^+^T_reg_ infiltrating HNSCC into six subsets, with subsets 2 and 4 representing early-stage CD4^+^T_reg_ with upregulated IFN-related signaling pathways, while subsets 3 and 6 represented late-stage CD4^+^T_reg_ with upregulated TNF-related signaling pathways (42). Guo et al. observed high expression of *Foxp3*, *IL-2RA*, and *IKZF2* in tumor-infiltrating CD4^+^T_reg_, as well as increased expression of *CTLA4*, *TIGIT*, and *TNFRSF9* in tumor-infiltrating CD4^+^T_reg_ in NSCLC specimens. Further investigation revealed that the TNFRSF9^+^subset within tumor-infiltrating CD4^+^T_reg_ exhibited potent immunosuppressive abilities (43). Azizi et al. identified five subsets of tumor-infiltrating CD4^+^T_reg_ in samples of breast cancer and analyzed the expression of immune checkpoint-related genes within each subset. The results showed that all subsets exhibited high expression of *CTLA4* and *GITR*, with three subsets showing elevated expression of *TIGIT (* 6). The widespread adoption of single-cell sequencing technology enables a more comprehensive analysis to the expression of CD4^+^T_reg_-related genes in tissue microenvironment, facilitating the identification of biomarkers that effectively represent CD4^+^T_reg_ functionality. However, these findings still require further validation through functional experiments.

## 5 Clinical trials targeting CD4^+^T_reg_ in cancer

Targeting CD4^+^T_reg_ has the potential to become an important approach in immunotherapy as tumor cells in TME can evade immune surveillance by recruiting immune-suppressive cells such as CD4^+^T_reg_. Considering the functional subgroups of CD4^+^T_reg_, the primary challenge lies in selectively targeting immunosuppressive subsets while minimizing the impact on other subpopulations, thus reducing the potential for undesirable autoimmune reactions. Early clinical trials primarily targeted CD25 as a marker for T_reg_, drugs such as denileukin and daclizumab have been reported to reduce the proportion of CD4^+^CD25^+^T_reg_ and enhance the response rate to tumor vaccine (44). However, CD25 is not a specific marker to CD4^+^T_reg_, and the combination of CD25^-^targeted therapy with tumor vaccines can suppress the generation of tumor-specific T cells (45), limiting the application of CD25 antibodies.

CCR4 is predominantly expressed on the surface of eT_reg_ (46), and previous studies indicated that blocking CCR4 can inhibit the accumulation of eT_reg_ in TME (35). Therefore, targeting CCR4 has shown certain efficacy. Mogamulizumab, a monoclonal antibody targeting CCR4, has been approved by the FDA for the treatment of cutaneous T-cell lymphoma (47). A clinical trial combined mogamulizumab with PD-1 inhibitor (NCT02476123) found an objective response rate of 12% in various types of locally advanced/metastatic solid tumors. The combination significantly reduced the proportion of eT_reg_ in both peripheral blood and tumor-infiltrating lymphocytes (TIL). Moreover, the combination therapy increased the proportion of CD8^+^T cells in TME regardless of tumor response, indicating the potential of CCR4 targeting therapy (48).

Inhibition of immune checkpoint receptors including CTLA-4, TIM-3, and LAG-3 had shown promising effects in clinical trials or pre-clinical studies. CTLA-4 is a co-inhibitory molecule expressed on the surface of CD4^+^T_reg_ and negatively regulates T cell activation (49). Studies in patients with metastatic melanoma have shown that the CTLA-4 antibody ipilimumab can prolong overall survival, but it is also associated with severe adverse events in 10% to 15% of patients (50). Recent clinical trials have found that PD-1 antibody has a lower incidence of adverse events compared to ipilimumab(KEYNOTE-006, NCT01866319) (51), and combination of nivolumab and ipilimumab (CheckMate 067, NCT01844505) can further extend overall survival in patients with melanoma (52). The data from this trial suggest that CTLA-4 antibodies may potentially serve as adjunctive therapy to enhance the efficacy of PD-1 antibodies. TIM-3 is expressed on innate immune cells. Although TIM-3 is not a specific marker on the surface of CD4^+^T_reg_, recent studies have identified TIM-3^+^CD4^+^T_reg_ within TME played a crucial inhibitory role by inducing exhaustion in effector T cells and TIM-3^+^CD4^+^T_reg_ might be a potential therapeutic target (53). Combination therapy targeting TIM-3 and PD-1 mAb has already been tested in several solid tumor, and an ongoing Phase 1 clinical trial is currently evaluating the dosage and anti-tumor efficacy of a humanized anti-TIM-3 antibody in advanced solid tumors (NCT02817633) (54). LAG-3 is expressed on activated T cells including CD4^+^T_reg_ (55) and LAG-3 can hinder the proliferation of effector T cells and dendritic cells, while promoting the differentiation of eT_reg_ (56). Currently, there are two clinical trials investigating the efficacy of anti-LAG-3 alone or in combination with PD-L1 antibody in patients with advanced solid tumors (NCT01968109, NCT03156114) (57). OX40 is a costimulatory molecule expressed mostly on activated effector T cells and nT_reg_ (58). Pre-clinical studies have demonstrated the immunosuppressive function of OX40^+^T_reg_ and anti-OX40 could improve tumor control in mouse models (59, 60). A recent phase I clinical in HNSCC patients (NCT02274155) demonstrated that anti-OX40 administration was well tolerated and increased the infiltration of activated CD4^+^ and CD8^+^T cells (61). This data indicates the potential clinical utility of anti-OX-40, but further clinical trials are needed to confirm its efficacy.

Above clinical trials aim to reverse the immunosuppressive state of TME by inhibiting immunosuppressive cells such as CD4^+^T_reg_. Optimization of combination therapies that effectively target CD4^+^T_reg_ while enhancing anti-tumor immune responses represents a promising avenue for the improved treatment of cancer. However, it is crucial to take into account the pathological subtype of cancer and adopt a targeted approach towards suppressive CD4^+^T_reg_, rather than indiscriminately depleting all T_reg_ or other effector T cells. These considerations highlight the importance of further evaluation when developing novel therapeutic strategies in this context.

## 6 Conclusion

Although CD4^+^T_reg_ represent a small proportion of lymphocytes, their immunoregulatory role in the TME is crucial. Effectively distinguishing the distinct functional heterogeneity of CD4^+^Treg subsets is a pressing challenge. Currently, the subtypes proposed by Sakaguchi et al. is the most widely used and exhibits good consistency in research conclusions. However, the subtypes of Th-like T_reg_ reflects the plasticity of CD4^+^T_reg_ in TME and still faces inconsistent findings across different studies. Furthermore, numerous clinical trials targeting CD4^+^T_reg_ have been conducted, highlighting the pressing need to accurately inhibit specific functional Treg subsets involved in immunoregulation. In future research, rational subgroup analysis based on different surface markers is essential for a better understanding of the role of CD4^+^T_reg_ in the TME.

## Author contributions

HL: Conceptualization, Project administration, Writing – original draft. YX: Conceptualization, Formal analysis, Writing – review & editing. CL: Conceptualization, Writing – review & editing.
